# Call for Action—Crisis Recovery and Preparedness in Child and Adolescent Public Health

**DOI:** 10.3389/phrs.2024.1606849

**Published:** 2024-05-09

**Authors:** Julia Dratva, Michal Molcho, Jean Calleja-Agius, Freia De Bock, Cecilia Elias, Marzia Lazzerini, Heiko Schmengler, Lourdes Cantarero-Arevalo, Emmanuelle Godeau, Danielle Jansen

**Affiliations:** ^1^ Department of Health Sciences, ZHAW Zürich University of Applied Sciences, Winterthur, Switzerland; ^2^ Medical Faculty, University of Basel, Basel, Switzerland; ^3^ School of Education, University of Galway, Galway, Ireland; ^4^ Faculty of Medicine and Surgery, University of Malta, Msida, Malta; ^5^ Department of General Pediatrics, Neonatology and Pediatric Cardiology, Medical Faculty and University Hospital Düsseldorf, Heinrich Heine University Düsseldorf, Duesseldorf, Germany; ^6^ National School of Public Health, Lisbon, Portugal; ^7^ Division of Infant, Youth, Reproductive and Sexual Health, Health Directorate General, Lisbon, Portugal; ^8^ Unidade de Saúde Pública Sintra, Sintra, Portugal; ^9^ Epitask Force—Faculdade de Medicina da Universidade de Lisboa, Lisboa, Portugal; ^10^ WHO Collaborating Centre for Maternal and Child Health, Institute for Maternal and Child Health—IRCCS “Burlo Garofolo”—Trieste, Trieste, Italy; ^11^ Maternal Adolescent Reproductive and Child Healthcare Centre, London School of Hygiene and Tropical Medicine, London, United Kingdom; ^12^ Department of Interdisciplinary Social Science, Faculty of Social and Behavioural Sciences, Utrecht University, Utrecht, Netherlands; ^13^ Faculty of Health and Medical Sciences, University of Copenhagen, Copenhagen, Denmark; ^14^ École des Hautes Etudes en Santé Publique (EHESP), Rennes, France; ^15^ Centre d’Epidémiologie et de Recherche en santé des Populations (CERPOP), Inserm UMR1295, Toulouse, France; ^16^ Department of Health Sciences, University Medical Center Groningen, University of Groningen, Groningen, Netherlands; ^17^ Department of Sociology, Interuniversity Center for Social Science Theory and Methodology (ICS), University of Groningen, Groningen, Netherlands

**Keywords:** child, adolescent, public health, crisis, health outcome

Europe is confronted with a series of crises: the COVID-19 pandemic, increased migration, the war in Ukraine and other global conflicts, energy shortage, and increased cost-of-living, all of which put achievements in child and adolescent public health at risk. Such events have aggravated existing health problems and increased inequality in health and wellbeing in general, but especially in children [1]. Children and adolescents, undergoing physical, mental and social developments, are particularly vulnerable to each crisis, let alone to the culmination of a number of subsequent and concurrent events, especially on the backdrop of a global climate crisis. While some crises do not have a direct effect on health, they impact most social determinants of health, hence indirectly affect health and wellbeing. Their concurrence most likely leads to adverse interactions and multiplicative adverse effects on health.

Child and adolescent health is defined as “the extent to which individual children or groups of children are able or enabled to a) develop and realize their potential, b) satisfy their needs and c) develop the capacities that allow them to interact successfully with their biological, physical, and social environments” [2]. Crises, irrespective of their origin, limit children’s and adolescents’ ability to flourish and reach their full potential, putting at risk the ability to meet their needs. At the same time, children and adolescents are an underrepresented group in society with limited power and voice, a fact which is aggravated by their marginalization in an aging society.

Crises disproportionately affect the most vulnerable, and risk to further decrease the resilience of young people, their families and communities [3, 4]. To address the preparedness for future crises and the most pressing public health problems for children and adolescents, the European Public Health Association (EUPHA) Child and Adolescent Public Health Section proposes the following actions to counteract the adverse health consequences of crises and to promote healthy environments, health and resilience among children and adolescents.

## Action 1: Increase Preparedness to Ensure Children’s Health in Times of Crises

Europe was not prepared for the most recent crises, particularly regarding protection and support of younger populations. The negative health consequences have been amply documented for the COVID-19 crisis [3, 4] while other crises are less well investigated. For a prepared and sustainable society, public health decision-making processes are needed that 1) are more inclusive to any vulnerable group, such as children and adolescents, 2) systematize in a transparent way the best available knowledge from all disciplines on all consequences (i.e., effects and side effects; short and long-term impacts) of potential public health decisions, 3) consult the younger populations (and guardians) as any other stakeholder and, 4) provide public services that protect fundamental rights of children and youth, e.g., safety from violence, having a home and minimal financial support, remain available in times of crises and anticipate emerging needs.

## Action 2: Data for Evidence-Based Actions

Effective, evidence-based interventions supporting child health and development as well as child healthcare delivery need to be scaled up. For this, and further to capture the relevance of any shifts at policy level, in health systems, or health and health behaviors of children and adolescents, data and evidence on social and environmental health determinants, child health outcomes and effective interventions are needed [5]. This means several critical points: 1) disaggregating the data to capture the developmental issues of childhood and adolescence; 2) adapting survey instruments to the targeted population to make sure the youngest as well as those with limited literacy or learning disorders can accurately give their true opinions; 3) including children and adolescents in building questionnaires and in choosing relevant topics from their perspective; 4) sharing the results with young people (and guardians) in a meaningful way.

## Action 3: Tackle Health Inequalities

All crises have affected, and will continue to affect, vulnerable and marginalized populations more strongly, and further exacerbate health inequalities. Inequalities in child and adolescent health are best addressed by focusing on social determinants of health, proportionate universalism (i.e., delivery of universal services at a scale and intensity proportional to the degree of need), and by using a multisectoral approach based on the principle of “Health in all Policies.” Furthermore, more equitable fiscal and labor market policies, which were proven to decrease inequality [6], are necessary to address the growing economic inequality in Europe and to lift up the 24.4% of children who were at risk of poverty and social exclusion across the European Union in 2021 [7]. It is likely that their number has increased further since the beginning of the war in Ukraine, and the associated cost-of-living crisis across the continent.

## Action 4: Strengthen Advocacy

In children, infant and under-5 mortality rates are typically utilized to monitor and assess population health; data are usually available and of high quality. However, child health determinants and outcomes in ages above 5 are most often not equitably assessed. The comparatively low infant and under-5 mortality rates in Europe can lead decision-makers to the conclusion that: *“All is well with child health*.*”* Advocacy for child health from 0–18 years is thus crucial to ensure decision-makers are aware of child and adolescent health issues, the long-term relevance, as well as of their responsibility to develop and implement policies and programs and to provide available services with sufficient funding. Advocacy should similarly focus on creating healthy environments for families and community support and to inform the public and opinion leaders about child and adolescent health and wellbeing issues and needs [8].

## Action 5: Increase Community Empowerment and Participation

A growing body of evidence is showing that increasing child and adolescent participation in public health research, policies, decisions and actions yields more positive and sustainable health outcomes, enhances participants’ empowerment and satisfaction, and also increases evidence due to improved data collection and dissemination of results [9]. Empowerment and participation of younger generations in public health actions and policy must guarantee diversity among the representatives, e.g., in terms of gender, age, cultural, socioeconomic and migrant backgrounds, cognitive and physical disabilities as well as sexual expression, and thus contribute to the development of a robust child and adolescent community. Participation trains young people to engage in decisions that matter to them with benefits in self-efficacy and self-worth [9], and also challenges and educates the adult community working in public health in terms of accepting shifting power dynamics.

### Conclusion

As child and adolescent public health experts, we observe considerable crises-related impact on core child and adolescent public health priorities [10]. A new vision is needed to support children and their families to recover from the negative consequences of the past and current crises and further to go stronger through new crises. This includes empowerment and participation of children and adolescents, improving the database on child health and effective interventions, addressing values, norms and traditions of children and their families, and protecting their fundamental rights. Further, health inequalities and needs of vulnerable young populations should be prioritized. An integrated and holistic approach and investments into children’s futures must be ensured through a comprehensive, long-term funding settlement, keeping in mind how needs change. The former World Health Organisation (WHO) child and adolescent health strategy “Investing in children: child and adolescent health strategy for Europe 2015–2020” has expired, a new strategy is pressingly needed. Crisis recovery and preparedness from a child and adolescent point of view is a central and urgent action, in which all policymakers, professionals of all sectors and disciplines, and young populations, must participate.
